# Dynamic adaptation of mesenchymal stem cell physiology upon exposure to surface micropatterns

**DOI:** 10.1038/s41598-019-45284-y

**Published:** 2019-06-24

**Authors:** Nick R. M. Beijer, Zarina M. Nauryzgaliyeva, Estela M. Arteaga, Laurent Pieuchot, Karine Anselme, Jeroen van de Peppel, Aliaksei S. Vasilevich, Nathalie Groen, Nadia Roumans, Dennie G. A. J. Hebels, Jan de Boer

**Affiliations:** 1 Department of Cell Biology Inspired Tissue Engineering, MERLN Institute for Technology-Inspired Regenerative Medicine, Maastricht University, Maastricht, The Netherlands; 2 Materiomics b.v., Maastricht, The Netherlands; 3 BioInterface Science lab, Department of Biomedical Engineering, Eindhoven University of Technology, Eindhoven, The Netherlands; 4 Institut de Sciences des Materiaux de Mulhouse, University of Haute-Alsace, CNRS UMR7361, Mulhouse, France; 5 Department of Internal Medicine, Erasmus University Medical Center, Rotterdam, The Netherlands

**Keywords:** Mesenchymal stem cells, Microarray analysis, Cytoskeleton, Cell division, Cell adhesion

## Abstract

Human mesenchymal stem (hMSCs) are defined as multi-potent colony-forming cells expressing a specific subset of plasma membrane markers when grown on flat tissue culture polystyrene. However, as soon as hMSCs are used for transplantation, they are exposed to a 3D environment, which can strongly impact cell physiology and influence proliferation, differentiation and metabolism. Strategies to control *in vivo* hMSC behavior, for instance in stem cell transplantation or cancer treatment, are skewed by the un-physiological flatness of the standard well plates. Even though it is common knowledge that cells behave differently *in vitro* compared to *in vivo*, only little is known about the underlying adaptation processes. Here, we used micrometer-scale defined surface topographies as a model to describe the phenotype of hMSCs during this adaptation to their new environment. We used well established techniques to compare hMSCs cultured on flat and topographically enhanced polystyreneand observed dramatically changed cell morphologies accompanied by shrinkage of cytoplasm and nucleus, a decreased overall cellular metabolism, and slower cell cycle progression resulting in a lower proliferation rate in cells exposed to surface topographies. We hypothesized that this reduction in proliferation rate effects their sensitivity to certain cancer drugs, which was confirmed by higher survival rate of hMSCs cultured on topographies exposed to paclitaxel. Thus, micro-topographies can be used as a model system to mimic the natural cell micro-environment, and be a powerful tool to optimize cell treatment *in vitro*.

## Introduction

Human mesenchymal stem or stromal cells (hMSCs) have a spherical morphology *in vivo and* display a mostly quiescent phenotype^1^. hMSCs are exposed to flat tissue culture polystyrene (TCP) the moment they are harvested from the human body and cultured *in vitro*. The cells rapidly adhere and spread to this two dimensional plane, and are exposed to cell culture medium containing fetal bovine serum. Under these circumstances, hMSCs are defined as plastic-adherent clonogenic cells which are multipotent, express CD73, CD90 and CD105 on their cell plasma membrane and lack expression of CD34, CD45, CD11b, CD14 and CD79^2^. hMSCs lack expression of telomerase and the majority of the bone marrow derived hMSC population is β-galactosidase positive after approximately 25 population doublings, indicating that they have reached replicative senescence and thus lack self-renewal capacity^3^. During *in vitro* cultivation, hMSCs also change their cell morphology towards higher surface area, and lose multipotency^4^. For this reason, hMSCs are used for *in vitro* experimental work typically within the first 5 passages, and in this window, their response to a wide variety of small molecules and cytokines is known, a clear example of which is the elevated expression of alkaline phosphatase upon incubation with dexamethasone^5^.

The influence of culture conditions on cell behavior is notorious. For instance, clear differences in proliferation rate and differentiation capacity are closely monitored when a new batch of serum is purchased^6^. In addition, there is growing awareness of the effect of the cell culture substrate on cell behavior. Here, substrates are explored which differ from TCP in both chemical and physical appearance in order to more closely mimic the *in vivo* situation. For example, hydrogels are orders of magnitude softer than TCP^7^, exotic mixtures of monomers can create unique chemical compositions^8^, and material surface structures can be modified on the nanometer-scale^9^ and micrometer-scale to provide cells a more physiological environment^10^. We and others have used micro-fabrication technologies to design and engineer surface topographies eliciting very defined cellular responses, which typically directly relate to the function of these cells in their tissue context. Depending on the type of surface topography and cell type, induced changes in cell behavior range from initiation of osteogenic differentiation of hMSCs^11^, adaptation of an anti-inflammatory M2 phenotype of macrophages^12,13^ or tissue formation of corneal epithelial cells^14^. In the margins of many scientific reports, it is reported that these functional phenotypes correlate to parameters which seem more inherent to the basic function of the cells, such as volume, shape, energy metabolism or granularity. For instance, multi-potency of hMSCs correlates to their size and metabolic profile^15,16^, and drug resistance of cancer cells is strongly correlated to their mitotic profile^17^. It is important to realise that most manuscripts provide detailed reports on functional phenotypes but mostly ignore these basic parameters, although it is known that microfabrication platforms are able to influence these basic phenotypes, as e.g. surface structure induced shifted cell cycle distribution^18^ and water flux controlled cell volume as a response to differential cell spreading^19^. In this manuscript, we set out to map surface topography induced changes in cellular state compared to hMSCs cultured on flat substrates. We followed the adaptation of hMSC phenotype within the first hours after contact up to a few days of culture, in terms of changes in cell and nucleus shape and volume, metabolism and cell cycle progression and documented a dramatic change in cell physiology over this period of time.

## Materials and Methods

### Topographically enhanced substrate production

TopoChip-derived surface topographies, selected based on topographical feature size and the cell morphology they induce, were placed in 15 mm circle format as the lay-out of a chromium masks for photolithography. Topographies used in this manuscript were patterns derived from the second generation TopoChip^10^, produced in polystyrene (PS). Topography nomenclature is based on the relative size of the topographical features, and is formulated as follows: Medium (M) = T2-PS-0304, Large (L) = T2-PS-1642, Small (S) = T2-PS-3240, and Extra Small (XS) = T2-PS-1901. T2 stands for the second TopoChip design as described in Unadkat *et al*., PS stands for polystyrene, the first two digits represent the row number counted from the top, and the second two digits represent the column number. The micrometer-scale patterns were etched from the silicon wafer by directional reactive ion etching (DRIE), generating a silicon master mould, and thus containing the inverse topography patterns. A three-replication process was used to fabricate the surface topography enhanced polystyrene films, using sequentially silicon, PDMS and Ormostamp moulds, as described before^20^. The Ormostamp moulds were for used for imprinting (Obducat Eitre 6 Nano Imprint Lithography system, Obducat, Sweden) into bi-axially oriented 190 µm thick polystyrene films (Goodfellow, United Kingdom) at 140 °C and 10 bar for 5 minutes. Untreated polished silicon wafers were used for embossing the unpatterned substrates. To increase hydrophilicity, we treated the topographically enhanced polystyrene films with a gentle O_2_-plasma (reactive ion etching home-build) at 10 °C, 50 sccm oxygen flow, 75 mTorr pressure and 50 W CCP power for 30 seconds). Prior to cell culture. all surfaces were sterilized using 70% ethanol for 1 hour, and were pre-treated with medium overnight before adding the hMSCs.

### Cell culture

For cell culture experiments, mesenchymal stem cells (hMSCs) from a human donor (female, 74 years-old) undergoing a total hip replacement were used. hMSCs were isolated from bone marrow after obtaining written informed consent from the patient. Ethical approval for using the bone marrow sample was obtained from the ethical advisory board of the Medisch Spectrum Twente, Enschede. All methods were carried out in accordance with local and relevant guidelines and regulations. hMSCs were expanded in basic hMSC medium consisting of α-minimal essential medium (a-MEM, Life Technologies) supplemented with 10% foetal bovine serum (FBS, Sigma), 2 mM L-glutamin (Fisher Scientific), 0.2 mM ascorbic acid (Sigma), 100 U/ml penicillin, and 100 mg/ml streptomycin (Fisher Scientific). To investigate the effect of surface topography on hMSCs (passage number 5), seeding densities of 10,000 cells/cm^2^ were used on both flat and topographically enhanced surfaces and grown for the designated times in a humidified incubator with 5% CO_2_ at 37 °C. Prior to experiments on cell size, protein synthesis, cell cycle distribution, proliferation, and chemotoxins, hMSCs were synchronized by serum depletion for 48 hours. hMSCs were incubated with paclitaxel (300–0.3 µM, Sigma) for 44 hours, starting with an initial cell density of 7,500 cell/cm^2^.

### Fluorescence staining and microscopy

For fluorescence microscopy, cells were fixated in freshly prepared 3.7% paraformaldehyde (Sigma) for 10 minutes at room temperature, permeabilized by 1% Triton-X (Sigma) in phosphate buffered saline (PBS) for 10 minutes and blocked for a-specific binding by 1% bovine serum albumin (BSA, Sigma Aldrich) in PBS for 30 minutes at room temperature. The actin cytoskeleton was stained with Phalloidin 488 (1:80, A12379 Thermo Fisher Scientific) for 40 minutes and DNA a stained with 4′,6-diamidino-2-phenylindole (DAPI, 14.3 µM, D1306 Invitrogen) for 5 minutes both in the dark and at room temperature. Epifluorescent micrographs were obtained using a Nikon A1 microscope, while confocal micrographs for nucleus volume quantification were obtained using a Leica SP8. For time-lapse imaging of fluorescently labeled U2OS cells (GFP-Actin) on topographically enhanced substrates, z-stacks of confocal images were taken every 5 minutes. Here, the three-dimensional reconstructed time-lapse confocal images were obtained using an upright Carl Zeiss LSM 700 with a humidified 5% CO_2_ chambers at 37 °C. Time resolved three-dimensional reconstructions of cells on topography M were created using Fiji^21^.

### Cell and nucleus size and shape analysis

Image analysis was performed using CellProfiler^22^. Analysis pipelines were customized for each dataset, and included background correction, cell identification and segmentation, and measurements on shape and size. We highlighted two shape descriptors, extent and eccentricity to quantify cellular and nuclear deformation. Cells with a relatively large extent, i.e. segmented cell area divided by the area of the bounding box, are circular or elliptical and have no protrusions. Nuclei with eccentricity (ratio of the distance between the foci of the ellipse and its major axis length) of 0 represent a circle, while an eccentricity of 1 represents a line. The volumes of the nuclei were quantified using FIJI, and based on the measurements of the three dimensional reconstructions. To quantify cell volume, hMSCs we trypsinized after 24 hours of culture, and quickly washed before direct measurements in the flow cytometer (BD Accuri C6). Cell size was measured using the forward scatter.

### Gene expression analysis

hMSCs were cultured for 7 days on the topographies and flat control surface before total RNA was isolated after a freeze thaw cycle using the Nucleospin RNA isolation kit (MarchereyNagel) according to the manufacturers protocol. cRNA was synthesized from 350 ng RNA using the Illumina TotalPrep RNA Amplification Kit. RNA and cRNA quality was verified on a Bioanalyzer 2100 (Agilent). The microarray analysis was performed using HT-12 v4 expression Beadchips (Illumina). According to the manufacturer’s protocol, 750 ng of cRNA was hybridized on the array overnight and the fluorescent signal was developed by adding streptavidin Cy-3. The bead chips were scanned on an Illumina Beadarray reader and the measured raw intensity values were background corrected in BeadStudio (Illumina). Further data processing and statistical testing was performed in R^23^ (R version 3.3.2 (2016-10-31)) using the Bioconductor statistical software. The probe-level raw intensity values were quantile normalized and transformed using variance stabilization (VSN). A linear modeling approach with empirical Bayesian methods, as implemented in Limma package^24^, was applied for differential expression analysis of the resulting probe-level expression values. Genes were considered differentially expressed between flat and topography when p < 0.05 and absolute fold change >1.5. The list of differentially expressed genes which overlapped in the expression profiles on all three topographies relative to the flat control surface was used for functional classification using PANTHER (http://pantherdb.org/)^25^. Using this database, the gene ontology of the introduced genes were grouped by biological process. The raw and normalized gene expression data and associated metadata have been uploaded in the Compendium for Biomaterial Transcriptomics (PID: https://hdl.handle.net/21.12109/CBIT_StudyID_018) and ArrayExpress (accession number E-MTAB-7505).

### Metabolic activity

HMSCs cultured on the flat and topographically enhanced substrates were assessed for their metabolic activity after 1, 3, 5, and 7 days, using the Presto Blue assay (A13261 Invitrogen) according to the manufacturer’s protocol. In brief, at the designated time points we replaced basic hMSC culture medium by Presto Blue medium (1x concentrated in basic hMSC medium), and incubated for 1 hour at 37 °C in a humid environment. Equal amounts of supernatant were subsequently transferred to a black/black bottom 96-well plate (Nunc, Fisher Scientific) and followed by the quantification of the fluorescent signal measured at 590 nm using a plate reader (Perkin Elmer, Victor3). After 72 hours of culture on topography XS, mitochondria were stained by incubating the samples with 25 nM MitoTracker deep red FM (AF647, M22426 Invitrogen) in basic medium for 15 minutes at 37 °C. Subsequently, cells were collected from the substrates and measured directly using the flow cytometer (BD Accuri C6).

### Cell proliferation

Newly synthesized DNA was detected using a live cell incorporation kit (Click-iT™ EdU Alexa Fluor™ 488 Imaging Kit, C10337 Invitrogen) according to the manufacturer’s protocol. In brief, after cell cycle synchronization by serum depletion for 48 hours, hMSCs were seeded on the flat control surface and topography XS where they were allowed to reenter the cell cycle by addition of 10% serum in the culture medium. 10 µM EdU was incubated for 40 hours basic medium before cells where fixed with 3.7% paraformaldehyde for 15 minutes. Next, cells were stained using the Click-it reaction buffer, and counterstained using Hoechst before fluorescent imaging.

For cell cycle measurement, hMSCs where cultured on flat and topography XS for 24 hours and, after trypsinization, resuspended in ice cold MilliQ water. Subsequently, ice cold absolute ethanol was added to the cell suspension to obtain a 70:30 ethanol:MilliQ mixture, in which the cells were fixed for 1 hour at 4 °C while shaking. After fixation, cells were washed with PBS and resuspended in a mixture of propidium iodide and RNAse (FxCycle™ PI/RNase Staining Solution, F10797 Invitrogen) and incubated for 30 minutes at room temperature. Finally, the DNA content was quantified by flow cytometry (BD Accuri C6). Cell cycle distribution analysis was performed with the FlowJo software, using the univariate fitting model for DNA content.

### Statistical analyses

Experiments were carried out in triplicate with three independent samples. Bar-graphs represent the mean ± standard deviation. Boxplots include the median values, with the boxes covering the 1^st^ and 3^rd^ quantile, the whiskers the highest and lowest values, and the dots the outliers. Samples were compared using the students t-test, with P < 0.05. Statistics used during gene expression analysis are explained in detail in the respective subsection on gene expression profiling.

## Results

### Cells actively remodel their shape to adapt to surface topographies

**Figure 1:**
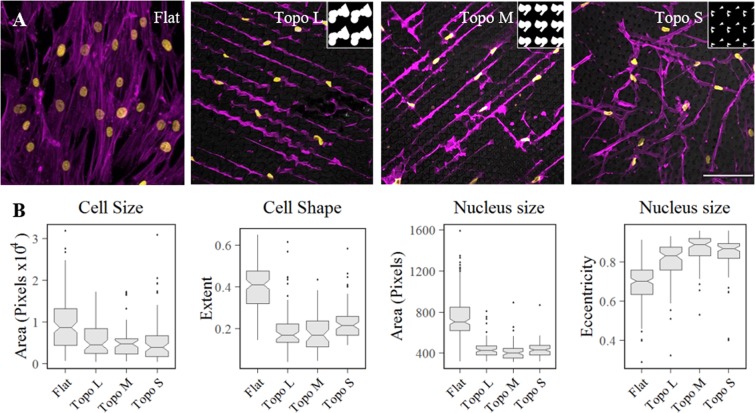
Surface topography induced changes in morphology (**A**) hMSCs cultured for 3 days on flat and topographically enhanced substrates (flat and topography M, L and S) show clear differences in both nuclear and cellular morphology. The inserts show the topographical feature designs of topography M,L and S, and each insert represents 50 by 50 µm. (**B**) Quantification of morphological descriptors Area and Extent for cells (N > 48), and Area and Eccentricity for nuclei (N > 75). For all measurements, the differences between Flat and the topographically enhanced substrates were found to be significant with p < 0.05. In the micrographs, the nuclei are stained in yellow using DAPI and the actin cytoskeleton in purple using phalloidin. The scale bar represents 100 µm.

Cell shape is the most eye-catching effect of micro-topography on cells and a clear example of cell adaptation. We selected three different TopoChip-derived topographies based on the confinement they will infer onto the cells. Besides a flat reference surface, we included substrates that were enhanced with large, medium, and small topographical features (see inserts Fig. 1A). After seeding, hMSCs mostly adhere in the valleys, and are thus surrounded by 10 µm high topographical features. hMSCs changed both their cell and nuclear morphology dramatically compared to the flat reference as seen after 3 days of culture (Fig. 1A). We observed strongly elongated cells with nuclei which seemed to be compressed and smaller in size. On topo M, cell shape was defined by long perpendicular structures, whereas cells on topo L tended to follow contact guidance into only one direction without branches. Cells on surface S however tended to produce structures into multiple directions with many branches. The width of the cell body correlated with the distance between the topographical features. Quantitative imaging of cells cultured on flat and the three topographies revealed a two-fold reduction of cell area as well as nuclear area (Fig. 1B).

**Figure 2:**
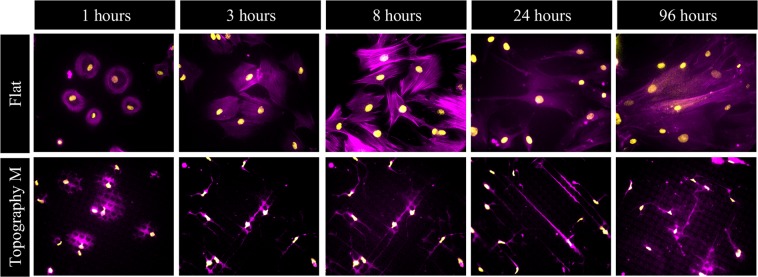
hMSCs adapt cell and nucleus morphology within the first hours after attachment to topographically enhanced substrates. hMSCs after 1, 3, 8, 24, and 96 hours of exposure to flat and topography M. The changes in cell and nucleus morphology between flat and topography are growing over time. Nuclei (yellow) are stained using DAPI and the actin cytoskeleton (purple) using phalloidin, the scale bar represents 50 µm.

To assess very early responses in terms of topography-induced cell shape deformation, and follow them over time, we seeded fluorescently labeled GFP-actin transgenic U2OS cells and imaged them from attachment for 24 hours on topography M (Fig. 1, video in Supplementary Fig. 1). As shown in supplemental video 1, globular cells in suspension touch the top of the topographical features but immediately start to descend to the valleys and start spreading within minutes. In video 2, one cell is followed for 5 hours and it is interesting to see how the cell continuously retracts and redirects cellular protrusions, as if it is sensing its environment. Throughout the imaging period, the cells of interest did not migrate, a trend that we confirmed for multiple other U2OS cells exposed to this surface topography. On the contrary, hMSCs did migrate on these topographically enhanced substrates (data not shown). Video 3 shows a dividing cell. First, the cell rounds up, sits on top of the topography and then divides to immediately spread and adhere again to the valley. Similar to U2OS cell, hMSCs actively adapt to their environment by constantly remodeling shape and size. A distinct difference in cell morphology between hMSCs on flat substrates and topographies is clearly visible after 1 hour (Fig. 2). Deformation increases over the course of the next 24 hours, when hMSCs displayed maximum spreading. Besides the changes in total cell morphology and size, cells also actively deform their nucleus within the same timeframe. The images demonstrate highly adaptive cells which remodel their shape relative to the micropattern to which they are exposed.

### Cells decrease nuclear and total cell volume under topographical confinement

**Figure 3:**
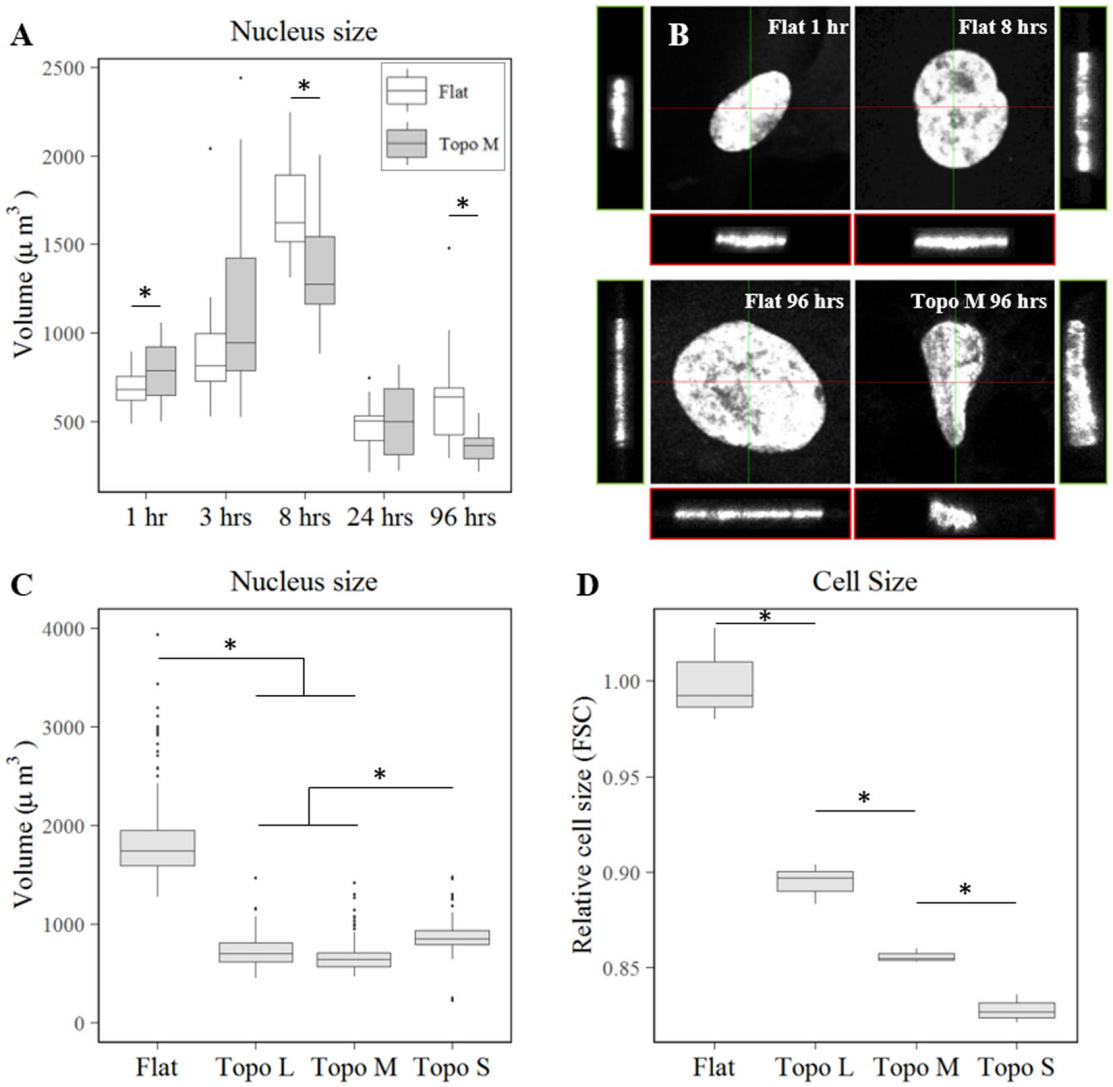
Topographical induced decrease in both nucleus and total cell size. (**A**) Dynamic change of nucleus volume (N > 11) in hMSCs cultured on flat and topography M after 1, 3, 8, 24, and 96 hours as quantified using confocal micrographs. (**B**) Volume views of representative nuclei after 1 and 8 hours on flat, and after 96 hours on flat and topography M. (**C**) Confocal micrograph quantification of nucleus volumes of cells on topographies with varying topographical feature size (S, M, and L) after 72 hours (N = 4). (**D**) Flow cytometer measurements of relative cell size after 72 hours on topographies with varying topographical feature size (S, M, and L) (N = 4). Statistical significance stars represent differences between conditions at a given time point with p < 0.05.

In previous work, we described that cells which are confined in the xy-plane become larger in the z-direction^11^. This suggests that confined cells change their shape, however, we assumed this would not give rise to a significant differences in total cellular or nuclear volume. To quantify the volumes of the nuclei we used three-dimensional (3D) reconstructions of cells cultured on flat and surface topographies using confocal imaging. In order to capture the dynamics of the deformation, we assessed nuclear volume after 1, 3, 8, 24 and 96 hours of exposure to flat and topography M (Fig. 3A). We observed a highly dynamic adaptation of the nuclear volume in which for both conditions, a two-fold increase in nuclear volume was observed during the first eight hours of culture. The next 16 hours, the nuclei shrunk significantly and volumes dropped three-fold for both conditions. At 96 hours, we observed a two-fold difference between the volumes of nuclei on flat substrates compared to topography M. The significant differences in nuclear volumes quantified for nuclei on flat substrates after 1 and 8 hours are due to reduced dimension in the xy-plane, and not from an decrease in the z-direction. The difference in nuclear volume between cells on flat and topography M after 96 hours did show an increase in the z-direction on topography, however, the difference in the xy-plane is significantly larger (Fig. 3B). Next, we wanted to see if such significant differences in nuclear volumes hold true for more topographies as well. Therefore, we assessed the deformation of the nuclei on the three topographies with varying topographical feature size (S, M, and L) after 72 hours of culture. Again, we observed a strong adaptation of cells on the different topographies. For all three assessed topographies, cells reduced the volumes of their nuclei up to 2.5-fold (Fig. 3C).

From the literature it is known that the size of the nucleus correlates with the size of the cytoplasm^26^. While the underlying mechanisms for this karyoplasmic ratio regulation are not completely elucidated yet, it is conserved across a wide variety of species. Since we observed that the cells decrease their nuclear size as a reaction to surface topographies, we wondered if this correlated with the total cell size. To assess total cell volume, we measured forward scatter by flow cytometry in cells harvested from the flat and topographically enhanced substrates. In line with the karyoplasmic ratio theory, we found that hMSCs significantly reduce their total cell volume after 72 hours of culture (Fig. 3D).

### Cells under confinement lower their metabolism

**Figure 4:**
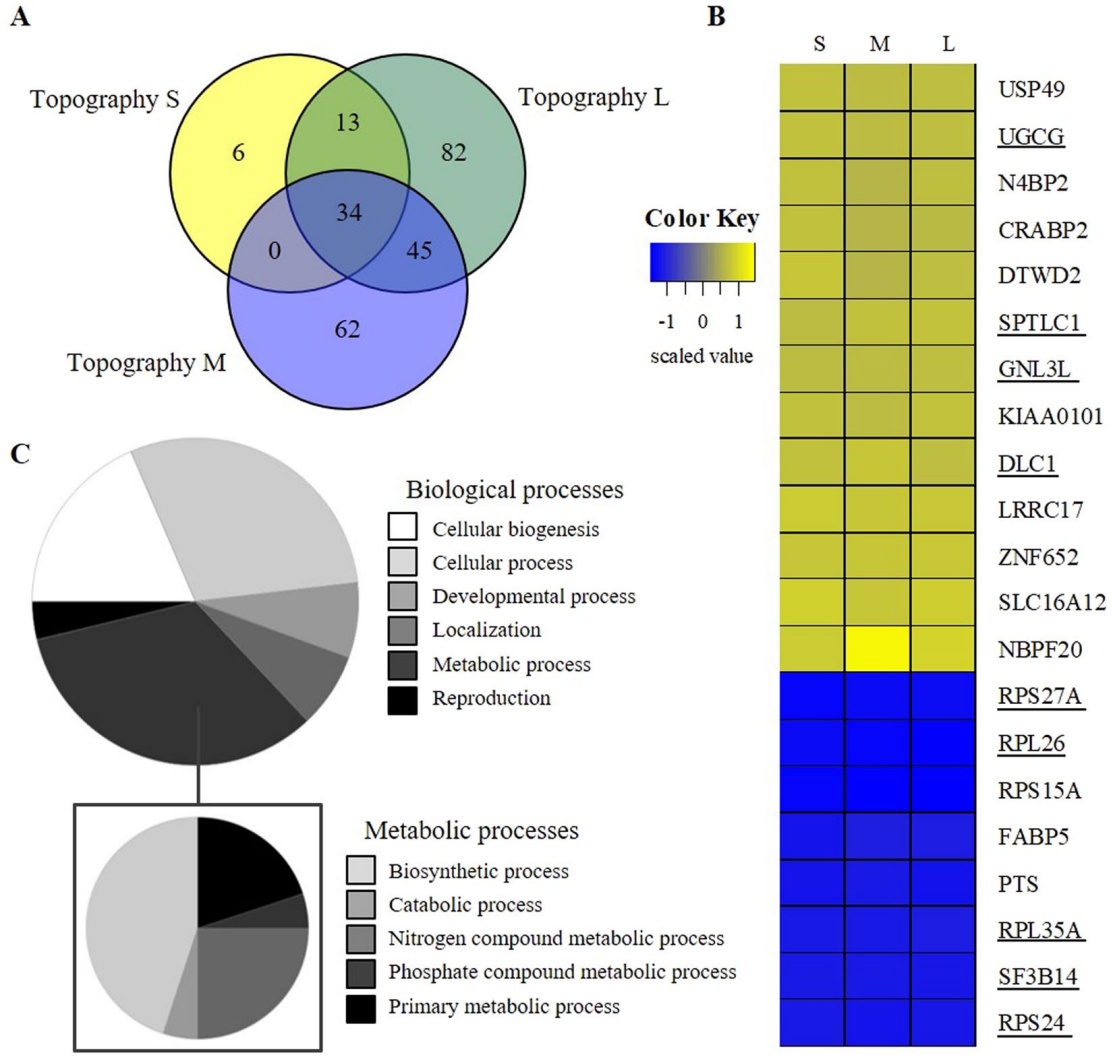
Topography induced differences in gene expression profiles. Microarray analyses of hMSCs after 7 days on topographically enhanced (S, M, and L) substrates compared to flat (N = 3). A) Venn diagram represents the number of DEGs which were unique for the topography conditions or which overlapped with the other condition(s). (**B**) Z-score scaled heatmap with DEGs, for each topography (S, M, and L), which were found in all three topographies. Underlined genes are involved in metabolic processes. (**C**) Proportional distribution representing the panther gene ontology classification analysis grouping the 34 DEGS to biological processes based on their gene ontology annotation. The list of DEGS linked to metabolic processes was specified further in a similar manner.

Next, we wondered whether nuclear deformation and sized were reflected in differential gene expression. For this, we compared the transcriptomics profiles of hMSCs cultured on flat and the three topographically enhanced substrates for seven days. The differentially expressed genes (DEGs) on the three topographies, compared to the flat reference, revealed genes which were unique for one specific topography, had an overlap with two topographies and DEGs found for all three topographies (Fig. 4A) relative to the unpatterned, flat substrate. The overlapping DEGs between the three topographies are thus typical for hMSCs which are adapting to a topographically enhanced microenvironment. Among the 34 overlapping DEGs there, 13 probes did not result in a protein product and were not included in further analysis. The genes showed all a similar trend in their expression compared to the flat reference conditions (Fig. 4B). Gene Ontology annotation of the overlapping DEGs revealed that 9 out of these 21 genes were related to metabolic processes (Fig. 4C). Among the 9 genes involved in metabolism, some encoded ribosomal proteins (RPS24, RPL26, and RPS27a) involved in protein synthesis, and in lipid metabolism (UGCG and SPTLC1). We found that the *Deleted in Liver Cancer-1* (DLC1) gene was expressed higher in cells cultured on topographies. As stated in the gene-name, this gene acts as a tumor suppressor since it inhibits cell growth and proliferation^27^. Besides liver cancer, it is involved in various other types of cancer, such as kidney, breast, lung, and prostate amongst others^28^. Furthermore, DLC1 activates GTP-bound GTPases to convert GTP into GDP (and thus inactivates them) in e.g. RhoA and Cdc42^29^. Elevated DLC1 levels as measured on topographies might therefore be associated with cytoskeleton organization and additionally, cell cycle regulation.

**Figure 5:**
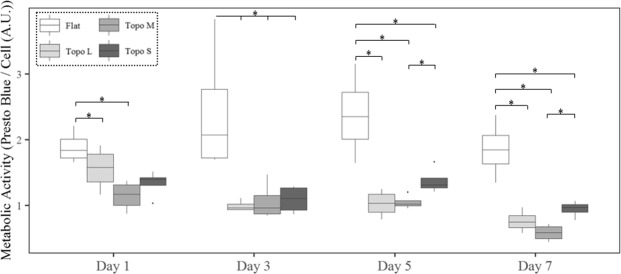
Topographical induced difference in metabolic activity develops over time. Mitochondrial activity, as quantified using Presto blue, differs significantly in hMSCs between flat and topography culture conditions (N = 4). Stars represent statistical significance P > 0.05.

To validate a change in metabolic activity, we exposed cells to non-fluorescent resazurin, which is converted into fluorescent resorufin in the reducing environment of mitochondria. Fluorescence intensities measured in this assay thus represent mitochondrial metabolic activity. Additionally, we quantified the number of cells to normalize metabolic activity. Using this method, we observed a lower metabolic activity already after 24 h on all three topographies relative to flat (Fig. 5). Where the metabolism of cells on flat surfaces remained constant, metabolism was lowered three-fold in cells exposed to topographies over a period of 7 days, suggesting a topography-dependent adaption process.

### Decrease in metabolic activity is not dependent on the degree of cellular confinement

**Figure 6:**
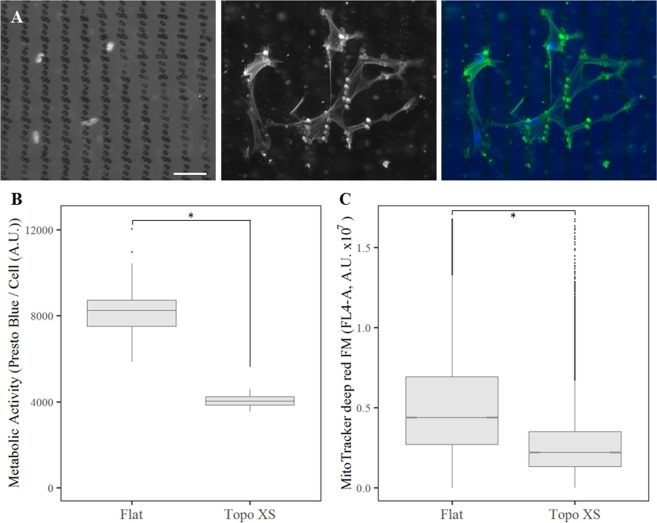
Mild cellular confinement leads to decreased metabolic activity. (**A**) hMSCs exposed for 72 hours to topography XS show mild confinement in morphology. Left shows DNA stained with DAPI, middle shows the actin cytoskeleton stained with phalloidin, right is a merge of both channels. Notes: left, one can appreciate the surface topographical features as darker spots. Middle, light captured from outside of the cellular boundaries comes from auto-fluorescence of the polystyrene. Scale bar represents 50 µm. (**B**) Presto Blue analysis for mitochondrial activity in hMSCs after 72 hours of culture on flat and topographically enhanced (XS) substrates (N = 5). (**C**) Flow cytometer quantification of the mitochondrial abundance in hMSCs cultured after 72 hours on topography XS compared to flat polystyrene (N = 3). In both **B** and **C** the differences between flat and topography XS are significant with p < 0.05, as indicated by the statistical significance star.

The phenotypic changes observed so far are induced by surface topographies that cause strong confinements for cells, as seen by the dramatically changed morphologies. Cells needed to adapt their total and nucleus morphology, and such strong deformations might have caused intense stress to which the cells adapt^30,31^. In order to assess the effect of a less confining topography on the degree of cellular adaptation, we selected a TopoChip-derived surface topography which allowed hMSCs to maintain their spread morphology. hMSCs grown on topography XS displayed a mild level of cell confinement when culturing hMSCs, which resulted in a classification ‘normal’ in a supervised clustering approach^32^ (Fig. 6A). Interestingly, even though the cells have the option to remain in the wide valleys between topographical features, we did occasionally observe strongly deformed nuclei indicating that the cells did migrate through the small pores. We also noted that cells used the topographies as anchor points.

Interestingly, hMSCs exposed to low confinement surface topographies still significantly reduced their overall cellular metabolism by a two-fold within three days (Fig. 6B). Because metabolism is measured as mitochondrial activity, we hypothesized this difference might depend on the number of mitochondria. We fluorescently stained the mitochondria in living cells and quantified the total number of mitochondria using flow cytometry. We observed a significant decreased in abundance of mitochondria in hMSCs exposed to topography XS for 3 days compared to hMSCs cultured on flat substrates (Fig. 6C).

### Cell cycle progression slows down in response to surface topography

**Figure 7:**
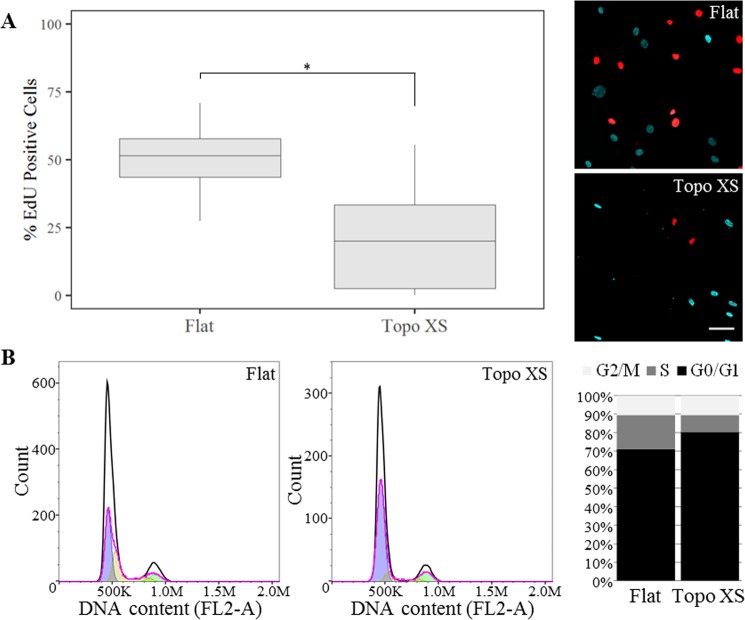
Surface topography inhibits cell cycle progression. (**A**) EdU-positive fraction of hMSCs exposed to flat and topography XS after 40 hours (N = 10), difference between conditions were significantly different with p < 0.05, as indicated by the statistical significance star. Representative micrographs showing the difference in quantity of EdU-positive cells on flat (top image) and surface topography (bottom image). Scale bar represents 100 µm. (**B**) Quantification of cellular DNA content (FL2-A) using flow cytometry revealed a shift in the cell cycle distribution between the flat and topography conditions after 24 hours of culture.

It is well-known that both cell and nuclear size increase during cell cycle progression^33^. While preparing for cell division, cells copy their DNA and multiply their organelles, which inevitably leads to an increase in volume^34^. However, cells cultured on topographically enhanced substrates are smaller and contain less organelles (in the form of mitochondria), and therefore we hypothesized a slower cell cycle progression. Consistent with the link between cell cycle progression and growing cell volume, we observed a 2.5-fold decrease in proliferation rates in the smaller cells cultured on surface topographies (Fig. 7A). We confirmed this observation in an different hMSC-donor, were we observed a comparable decrease in the proliferation rate on Topography M (Supplementary Fig. 2). Furthermore, assessment of the cell cycle distribution revealed a higher prevalence of cells in the G_0_/G_1_-phase accompanied by a decrease in the number of cells in S-phase for cells cultured on surface topographies (Fig. 7B). Interestingly, the fraction of the cell population which were in the G_2_/M-phase of both flat and topographically enhanced conditions remained the same.

### hMSCs on topographies are less sensitive to paclitaxel

**Figure 8:**
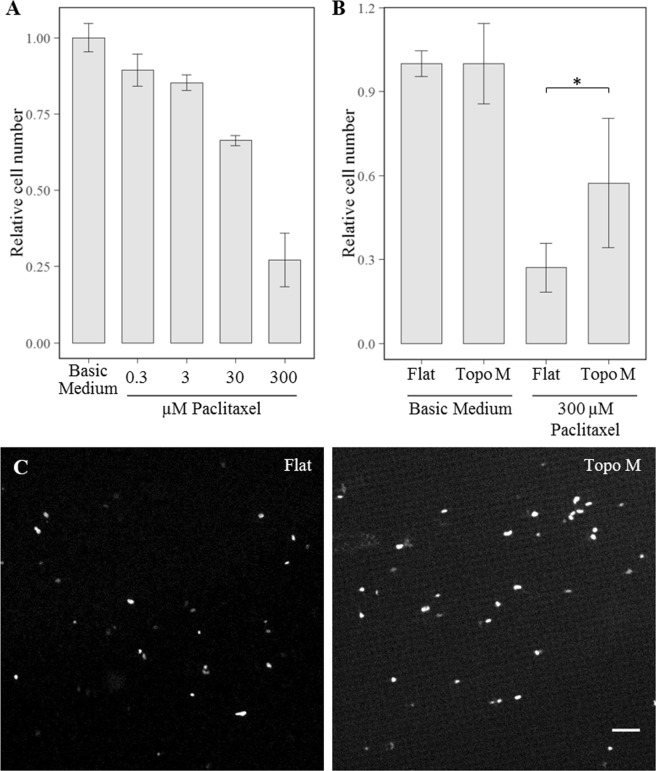
Surface topography adapted hMSCs are more resistant to anti-cancer drugs. (**A**) dose-response curve of MSC survival after a 44 hours paclitaxel treatment (N = 3). The difference between cell numbers for cells exposed to paclitaxel cultured on Flat and Topo M were found to be significantly different with p < 0.05. (**B**) Differential resistance to paclitaxel of MSC cultured on flat of topographically enhanced substrates after 44 hours. Cell counts are normalized to the basic medium condition. (**C**) Representative micrographs of nuclei staining after 44 hours of exposure to 300 µM paclitaxel on flat (left) and topography M (right). Scale bar represents 50 µm.

Cancer therapies often target metabolically active, dividing cells. The cancer drug paclitaxel increases stability of the microtubules and thus interferes with mitosis, resulting in G_2_/M cell cycle arrest and apoptosis^35,36^. Based on the lower level of metabolism and cell cycling, we reasoned that cells on topographies are less sensitive to paclitaxel. We exposed hMSCs for 44 hours to a range of paclitaxel concentrations and chose 300 µM paclitaxel, which resulted in a 75% reduction of cell number compared to hMSCs cultured in basic medium after 44 hours (Fig. 8A). Next, compared cell survival between a flat control surface and hMSCs cultures on topography M, and observed that two times more cells remained alive after the paclitaxel treatment when being exposed to surface topography (Fig. 8B,C). The effect of topography to paclitaxel sensitivity was further confirmed with the analogous microtubule-stabilizing drug docetaxel (data not shown).

## Discussion and Conclusion

**Figure 9:**
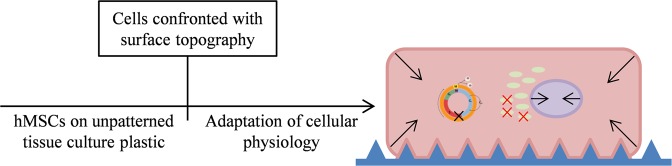
Topography induced cell behavior adaptation. After initial cell-surface topography contact, cells quickly respond and create an adapted cellular state. Here, we observe shrinkage of cell and nucleus sizes, condensation of proteins, reduction in mitochondria abundance, and cell cycle arrest.

The in house developed TopoChip platform is used for screening of algorithm generated surface topographies for their influence on cell phenotype, and typically in terms of differentiation^10^. However, besides differential expression of differentiation markers we often observed changes in other cellular functions as well compared to cells cultured on flat TCP. These changes occur upon cell attachment to topographically enhanced substrates, where cells become physically confined by the topographical features. Within the first 24 hours, cells adapt to their new environment and from there continue to reach equilibrium in their cellular state. Compared to cells that attach to flat tissue culture plastic, we observed topographically-induced changes in cell and nuclear morphology and volume, mitochondria abundance causing a lower overall metabolism; and reduced cell cycle progression (Fig. 9).

Cell-material interaction – from which the behavior controlling mechanotransduction arises – initiates upon cell attachment. As early as 1964, Adam Curtis used interference reflection microscopy to assess the shape of cells and the proximity towards the adhesive substrate after cellular attachment^37^. More recently, Pierres *et al*. showed – using a similar microscopy technique– that cells first attach with only small protrusions in a ‘tiptoe-like’ manner, after which complete cell attachment occurs during the following tens of seconds^38^. Other studies – in which biomaterials with modified surfaces are used – showed that contact guidance comes in play immediately after initial attachment, with fibroblasts elongating along linear patterned fibronectin within 30 minutes^39^. Furthermore, guided by surface structure, significant differences in morphology are typically observed within minutes to hours, with total cellular areas remaining similar between surface structure and flat conditions^40^. Using human osteosarcoma-derived cells (SaOs-2), Davidson *et al*. observed full deformation of both total cells and nucleus after 24 hours, but not yet after 6 hours, as a reaction to 7 by 7 µm sized 4 µm tall micropillars^41^. This is in line with our findings, in which we observed cellular deformation immediately after attachment and continued until around 24 hours later where we observed a maximum deformation state. Also for this period after the first 24 hours, it is known that both total cell and nuclear shape are constantly changing, with e.g. clear differences in elongation. Interestingly, the magnitude of deformation is found to decrease around 14 days of continuous exposure to micropillars^42^.

For nuclear deformation in particular, we observed an increase in nucleus size between 1 and 8 hours after initial attachment, on both flat and patterned surfaces. In terms of deformation (not volume), Lui *et al*. observed a maximum around 8 hours of exposure to micropillars, in line with our observed maximum volume. In the hours following, this deformation partially recovered^43^. Moreover, this recovery process did not complete and micropillar-induced deformations persisted over longer periods of time^44^. For mouse embryonic fibroblasts that detach from their substratum, it has been measured that both total cell and nucleus volumes reduce by 50%^45^. We hypothesize that the maximum nuclear volume, measured around 8 hours on our substrate, might be the end stage of nuclear volume increase that occurs when cells attach. It has to be noted that as seen for total cell morphology^46^, observed trends in nucleus deformation are dynamic and highly depended on cell type^47,48^.

Cell attachment and spreading are accompanied by water efflux, resulting in reduction of cell volume. This reduction in cell volume was found to be linearly correlated with their nucleus volume, and holds true for multiple cell types^19^, and seems to correlate to the strength of attachment. Spreading controlled reduction in cellular volume was amplified by our topographies with an extra 15% reduction in cell diameter after 24 hours. With cell deformation, as e.g. elongation, it is reported that the nucleus elongates as well, guided by the cytoskeletal organization, and reduces its volume^49^. In both examples from literature, cells attached to adhesion promoting surfaces which resulted in a maximum amount of cell spreading and reduction of cellular volume. In contrast, we observed lower adherence of hMSCs to patterned than flat surfaces, while cells had a smaller volume (manuscript in prep.). Based on this apparent contradiction, we hypothesize that in addition to adherence-dependent water efflux, there is an adherence-independent topography-induced mechanism that plays a role in regulating cell size as well.

With a reduction of both cytoplasm and nucleus the karyoplasmic ratio remains constant. Several theories exists on the control of this nucleus/cytoplasm ratio, however, thorough understanding of the involved mechanisms remain to be elucidated. For example, during dedifferentiation of matured cell types into stem cells it is known that the karyoplasmic ratio also increases (stem cells have a relatively small cytoplasm)^50^. In the current report, we did not present data on this ratio, however, extrapolation of the measured cell radii by flow cytometry revealed a 40% reduction in cell volume which approximates the 50% reduction of nucleus size. While the karyoplasmic ratio is described to be maintained in many occasions, like e.g. cell attachment^19^ and micropillar-induced deformation^40^, it is known that external stimuli can cause changes in this ratio^51^. Moreover, this regulation is known to be important, since disturbed karyoplasmic ratios are e.g. related to several types of cancer^52,53^.

In many cell types, the lowest nuclear volume is found directly after cell division, after which the cells start growing and DNA is replicated once S phase of the cell cycle has been entered. Furthermore, transcriptional activity of nuclei is correlated with larger nuclei^54^. Since many protein interactions are occurring in a stochastic manner, it is important to realise that nuclear deformation has an effect on local protein concentration. Here, the nuclear volume can be of great influence in terms of enabling interactions^55^. Also in deformed nuclei, genes can be differentially localized within the nucleus which can alter their availability for the transcription machinery^56^. Furthermore, evidence is found that mechanotransduction – by using dynamic loading on cells, without any exogenous differentiation factors – can induce the condensation of chromatin via acto-myosin mediated cytoskeletal tension^57^. Spatial organization of DNA is increasingly appreciated as an important contributor to genomic functions, and using 4C-technology (chromosome conformation capture on-chip) it has been observed that different DNA loci are interconnected. Within regularly shaped nuclei, these intrachromosomal interactions can occur at “long-ranges” within the nucleus^58^. The genes which are in contact with each other share transcription factors, which can lead to so-called variegated expression^59^. Higher-order chromosome structure is known to be important in the growth and development of organisms^60^, and in pluripotent stem cells e.g. it is known that there is a unique genome structure around pluripotency factor OCT4 and NANOG^61^. Especially in terms of chromosomal organization, topography-induced nuclear deformations may create great impact. Besides the large size reduction, the dramatic changes in nucleus morphology will strongly influence the spatial distribution of the chromosomes. Advanced techniques such as 4 C might provide us with more insight in the altered transcriptional activity and affected downstream cellular processes.

The differential activity and functionality in smaller nuclei holds also true for smaller cells^62^. Here, little is known about the mechanisms that regulate homeostasis of mammalian cell size. In human cells, Largen – a product of the PRR16-gene resulting in an increased cell size when overexpressed – was described by Yamamoto *et al*. to correlate with a higher mitochondrial activity^63^. In line with this, on our topographies we observed that smaller cells had a lower mitochondrial activity. Additionally – and also in line with our data on mitochondrial abundance – it is known that smaller cells contain fewer organelles^34^. However, also disagreeing reports exist on this topic, where transcriptomics analysis of cyclin-depended kinase 1 lacking cells revealed that an increase in cell size corresponded to decreased gene expression involved in mitochondrial function^64^. Without significantly altering hMSC size and shape, Mcmurray *et al*. observed changes in metabolic processes induced by surface structure. Here, ordered arrays of nanopits activate small RNAs which are in involved in repressing metabolic pathways, resulting in a longer maintenance of stemness^65^.

The reduced proliferation rates observed in cell populations exposed to our topographies can be linked to multiple mechanobiological molecular mechanisms. For example, YAP is a well-known mechanosensitive protein^66^, and strongly involved in tissue growth and proliferation^67^. YAP is part of the Hippo pathway, which is named after its involvement in developmental growth of organs where inhibiting the pathway results in tissue overgrowth^68^. In active canonical Hippo signaling – for example by cell-cell contact (contact inhibition) – LATS phosphorylates YAP, which results in sequestering of YAP in the cytoplasm and subsequently degradation^69^. Un-phosphorylated YAP – as a result of inactive Hippo – is translocated into the nucleus where it acts as co-transcription factor for many gene expression programs, including proliferation programs^68^. However, upon mechanical activation, YAP becomes nuclear active through non-canonical Hippo signaling. Here, for example cytoskeletal reorganization can play an important role^70^.

Besides YAP, Lee *et al*. found MAKP/ERK signaling to be activated in MSCs via mechanotransduction, by exposure to nanopit-substrates. Moreover, these MSC populations contained a larger fraction of cells in the G_0_/G_1_-phase. Here, the progression from the G_1_-phase was hypothesized to be inhibited via a cdc2-dependent mechanism, and repression of the S-phase transition by p27kip1 activity^18^. Surface structure-induced shifts in cell cycle distribution were found to be similar to our data. Accompanying increases in cell size is the chromatin decondensation mediated increase in nucleus size which strongly correlates with DNA synthesis^71^. In terms of the reduced nuclear volumes as found on our topographies, it has been described that smooth muscle cells with deformed nuclei barely proliferate. This reduction in proliferation rate is hypothesized to come from the deformation of mature lamin structures which might be exposed to higher internal stress^72^. In our transcriptomics data we found multiple ribosomal protein encoding genes with reduced expression. It is known that the ribosomal proteins are involved in p53-depentent metabolic regulation of cell cycle and cell growth via protein synthesis. Compared to cells cultured on flat substrates – with a high mitochondrial metabolism – ribosomal protein expression is down-regulated on topographies. This reduction in ribosomal proteins causes less inhibition of the p53-inhibitor Mdm2, and thus results in stabilization of p53 and lower level of mitochondrial metabolism^73^. This is in line with our hypothesis that cells *in vivo* have a lower energetic state compared to cells which are brought *in vitro* onto flat substrates, and which is undone when subsequently topographies are introduced and result in a lower metabolism. Furthermore, ribosomal proteins are found to activate p53-dependent cell cycle check points, and thus strongly involved in cell cycle regulation^74–76^.

Mechanotransduction plays an important role in cancer biology, with e.g. often a higher tissue stiffness found in tumors and strong migratory phenotypes of metastatic cells. In most cancers, fascin – an F-actin-bundling protein – is significantly upregulated, which correlates with poor clinical prognosis^77^. Furthermore, cisplatin-induced hair cell death is dependent on functional mechanotransduction in the zebrafish lateral line^78^. Activation of YAP is often observed in carcinomas, and resistance of tumor cells to chemotherapeutics correlates to YAP activity. For example, is it known that YAP activation in breast cancer cells can promote survival treatment to paclitaxel, and protects cancer cells against the effects of the DNA-damaging agents such as cisplatin^79^. In this work we used the anti-cancer drug paclitaxel as a compound which stabilizes the microtubules and by this causes a defect in the mitotic spindle function, resulting in apoptosis of those cells. There might be a role for the mechanosensitive protein YAP in the topographically-induced mechanotransduction-controlled resistance against paclitaxel as well. Having an *in vitro* cell culture system which is potent to control proliferation and metabolism might be a very interesting addition to experimental cancer research and toxicology. In this report, we demonstrate that topographically enhanced substrates reduce the sensitivity of MSCs to anti-cancer drugs in terms of cell viability after treatment. Therefore, topographies enable researchers to investigate mechanisms underlying the effects of anti-cancer drugs on cells in a chemically and genetically unchanged system.

As a result of a high metabolism and proliferation rate, metabolites and accompanying cellular stressors can accumulate within cells^80^. In this work, we describe how cells reduce their metabolism and proliferation rate when adapting towards a topographically enhanced cell culture substrate. This adapted phenotype might therefore lower the amount of metabolic stressors, and therewith lower the risk for developing malfunctioning cellular processes. Topographically enhanced substrates can thus serve as model system for the study of very basal cell function involved in cancer and ageing.

## Supplementary information

Supplementary figures

## References

[1] Nombela-Arrieta C, Ritz J, Silberstein LE (2011). The elusive nature and function of mesenchymal stem cells. Nat Rev Mol Cell Biol.

[2] Dominici M (2006). Minimal criteria for defining multipotent mesenchymal stromal cells. The International Society for Cellular Therapy position statement. Cytotherapy.

[3] Alves H (2010). A link between the accumulation of DNA damage and loss of multi-potency of human mesenchymal stromal cells. J. Cell. Mol. Med..

[4] Alves H, Mentink ALB, van Blitterswijk CA, de Boer J (2013). Effect of antioxidant supplementation on the total yield, oxidative stress levels, and multipotency of bone marrow-derived human mesenchymal stromal cells. Tissue Eng Part A.

[5] Jaiswal N, Haynesworth SE, Caplan AI, Bruder SP (1997). Osteogenic Differentiation of Purified, Culture-Expanded Human Mesenchymal Stem Cells *In Vitro*. J. Cell. Biochem..

[6] Hemeda H, Giebel B, Wagner W (2014). Evaluation of human platelet lysate versus fetal bovine serum for culture of mesenchymal stromal cells. Cytotherapy.

[7] Mosiewicz KA (2013). *In situ* cell manipulation through enzymatic hydrogel photopatterning. Nat. Mater..

[8] Mei Y (2010). Combinatorial development of biomaterials for clonal growth of human pluripotent stem cells. Nat. Mater..

[9] Dalby Matthew J., Gadegaard Nikolaj, Tare Rahul, Andar Abhay, Riehle Mathis O., Herzyk Pawel, Wilkinson Chris D. W., Oreffo Richard O. C. (2007). The control of human mesenchymal cell differentiation using nanoscale symmetry and disorder. Nature Materials.

[10] Unadkat HV (2012). An algorithm-based topographical biomaterials library to instruct cell fate. Proc. Natl. Acad. Sci..

[11] Hulshof FFB (2017). Mining for osteogenic surface topographies: In silico design to *in vivo* osseo-integration. Biomaterials.

[12] Luu TU, Gott SC, Woo BWK, Rao MP, Liu WF (2015). Micro and Nano-patterned Topographical Cues for Regulating Macrophage Cell Shape and Phenotype. ACS Appl Mater Interfaces.

[13] Sridharan R, Cameron AR, Kelly DJ, Kearney CJ, O’Brien FJ (2015). Biomaterial based modulation of macrophage polarization: A review and suggested design principles. Mater. Today.

[14] Karuri NW (2004). Biological length scale topography enhances cell-substratum adhesion of human corneal epithelial cells. J. Cell Sci..

[15] Bijonowski, B. M., Daraiseh, S. I., Yuan, X. & Ma, T. Size-dependent Cortical Compaction Induces Metabolic Adaptation in Mesenchymal Stem Cell Aggregates. *Tissue Eng Part A* **0** (2018). 10.1089/ten.tea.2018.0155 PMC6482905 30187829

[16] Liu Y, Munoz N, Bunnell BA, Logan TM, Ma T (2015). Density-Dependent Metabolic Heterogeneity in Human Mesenchymal Stem Cells. Stem Cells.

[17] Shah MA, Schwartz GK (2001). Cell cycle-mediated drug resistance: An emerging concept in cancer therapy. Clin. Cancer Res..

[18] Lee LCY (2017). Nanotopography controls cell cycle changes involved with skeletal stem cell self-renewal and multipotency. Biomaterials.

[19] Guo M (2017). Cell volume change through water efflux impacts cell stiffness and stem cell fate. Proc. Natl. Acad. Sci..

[20] Zhao Y (2017). High-definition micropatterning method for hard, stiff and brittle polymers. Mater. Sci. Eng. C.

[21] Schindelin J (2012). Fiji: An open-source platform for biological-image analysis. Nat. Methods.

[22] Carpenter AE (2006). CellProfiler: image analysis software for identifying and quantifying cell phenotypes. Genome Biol..

[23] R Core team. R. A Language and environment for statistical Computing. (2014).

[24] Ritchie Matthew E., Phipson Belinda, Wu Di, Hu Yifang, Law Charity W., Shi Wei, Smyth Gordon K. (2015). limma powers differential expression analyses for RNA-sequencing and microarray studies. Nucleic Acids Research.

[25] Thomas PD (2003). PANTHER: A library of protein families and subfamilies indexed by function. Genome Res..

[26] Jorgensen P (2007). The Size of the Nucleus Increases as Yeast Cells Grow. Mol. Biol. Cell.

[27] Zhou X, Thorgeirsson SS, Popescu NC (2004). Restoration of DLC-1 gene expression induces apoptosis and inhibits both cell growth and tumorigenicity in human hepatocellular carcinoma cells. Oncogene.

[28] Liao Y-C, Lo SH (2009). Deleted in Liver Cancer-1 (DLC-1): a tumor suppressor not just for liver. Int J Biochem Cell Biol..

[29] Kim TY (2007). DLC-1, a GTPase-activating protein for Rho, is associated with cell proliferation, morphology, and migration in human hepatocellular carcinoma. Biochem. Biophys. Res. Commun..

[30] Denais CM (2016). Supplementary Materials for Nuclear envelope rupture and repair during cancer cell migration. Science (80-.)..

[31] Gauthier NC, Masters TA, Sheetz MP (2012). Mechanical feedback between membrane tension and dynamics. Trends Cell Biol..

[32] Beijer NRM (2017). TopoWellPlate: A Well-Plate-Based Screening Platform to Study. Cell – Surface Topography Interactions. Adv. Biosyst..

[33] Echave P, Conlon IJ, Lloyd AC (2007). Cell size regulation in mammalian cells. Cell Cycle.

[34] Schmoller KM, Skotheim JM (2015). The Biosynthetic Basis of Cell Size Control. Trends Cell Biol..

[35] Bacus SS (2001). Taxol-induced apoptosis depends on MAP kinase pathways (ERK and p38) and is independent of p53. Oncogene.

[36] Bharadwaj R, Yu H (2004). The spindle checkpoint, aneuploidy, and cancer. Oncogene.

[37] Curtis ASG (1964). The Mechanism of Adhesion of Cells to Glass: A Study by Interference Reflection Microscopy. J. Cell Biol..

[38] Pierres A, Benoliel AM, Touchard D, Bongrand P (2008). How cells tiptoe on adhesive surfaces before sticking. Biophys. J..

[39] Ramirez-San Juan GR, Oakes PW, Gardel ML (2017). Contact guidance requires spatial control of leading-edge protrusion. Mol. Biol. Cell.

[40] Sales A, Holle AW, Kemkemer R (2017). Initial contact guidance during cell spreading is contractility-independent. Soft Matter.

[41] Davidson PM, Özçelik H, Hasirci V, Reiter G, Anselme K (2009). Microstructured surfaces cause severe but non-detrimental deformation of the cell nucleus. Adv. Mater..

[42] Hasturk O (2016). Quantification of Type, Timing, and Extent of Cell Body and Nucleus Deformations Caused by the Dimensions and Hydrophilicity of Square Prism Micropillars. Adv. Healthc. Mater..

[43] Liu X, Liu R, Gu Y, Ding J (2017). Nonmonotonic Self-Deformation of Cell Nuclei on Topological Surfaces with Micropillar Array. ACS Appl. Mater. Interfaces.

[44] Liu X (2016). Subcellular cell geometry on micropillars regulates stem cell differentiation. Biomaterials.

[45] Kim D-H (2016). Volume regulation and shape bifurcation in the cell nucleus. J. Cell Sci..

[46] Davidson PM (2010). Topographically induced self-deformation of the nuclei of cells: Dependence on cell type and proposed mechanisms. J. Mater. Sci. Mater. Med..

[47] Badique F (2013). Directing nuclear deformation on micropillared surfaces by substrate geometry and cytoskeleton organization. Biomaterials.

[48] Ermis M, Akkaynak D, Chen P, Demirci U, Hasirci V (2016). A high throughput approach for analysis of cell nuclear deformability at single cell level. Sci. Rep..

[49] Versaevel M, Grevesse T, Gabriele S (2012). Spatial coordination between cell and nuclear shape within micropatterned endothelial cells. Nat. Commun..

[50] Cai S, Fu X, Sheng Z (2007). Dedifferentiation: A New Approach in Stem Cell Research. Bioscience.

[51] Swanson JA, Lee M, Knapp PE (1991). Cellular dimensions affecting the nucleocytoplasmic volume ratio. J. Cell Biol..

[52] Slater DN (2005). Proposed Sheffield quantitative criteria in cervical cytology to assist the grading of squamous cell dyskaryosis, as the British Society for Clinical Cytology definitions require amendment. Cytopathology.

[53] Johnston DG (1952). Cytoplasmic nuclear ratios in the cytological diagnosis of cancer. Cancer.

[54] Webster M, Witkin KL, Cohen-Fix O (2009). Sizing up the nucleus: nuclear shape, size and nuclear-envelope assembly. J. Cell Sci..

[55] Misteli T (2008). Nuclear order out of chaos. Nature.

[56] Anselme K, Wakhloo NT, Rougerie P, Pieuchot L (2017). Role of the Nucleus as a Sensor of Cell Environment Topography. Adv. Healthc. Mater..

[57] Heo SJ (2015). Biophysical regulation of chromatin architecture instills a mechanical memory in mesenchymal stem cells. Sci. Rep..

[58] Simonis M (2006). Nuclear organization of active and inactive chromatin domains uncovered by chromosome conformation capture-on-chip (4C). Nat. Genet..

[59] Noordermeer D (2011). Variegated gene expression caused by cell-specific long-range DNA interactions. Nat. Cell Biol..

[60] De Laat W, Duboule D (2013). Topology of mammalian developmental enhancers and their regulatory landscapes. Nature.

[61] De Wit E (2013). The pluripotent genome in three dimensions is shaped around pluripotency factors. Nature.

[62] Lloyd AC (2013). The regulation of cell size. Cell.

[63] Yamamoto K (2014). Largen: A Molecular Regulator of Mammalian Cell Size Control. Mol. Cell.

[64] Miettinen TP (2014). Identification of transcriptional and metabolic programs related to mammalian cell size. Curr. Biol..

[65] Mcmurray RJ (2011). Nanoscale surfaces for the long-term maintenance of mesenchymal stem cell phenotype and multipotency. Nat. Mater..

[66] Dupont S (2011). Role of YAP/TAZ in mechanotransduction. Nature.

[67] Huang J, Wu S, Barrera J, Matthews K, Pan D (2005). The Hippo signaling pathway coordinately regulates cell proliferation and apoptosis by inactivating Yorkie, the Drosophila homolog of YAP. Cell.

[68] Hansen CG, Moroishi T, Guan KL (2015). YAP and TAZ: A nexus for Hippo signaling and beyond. Trends Cell Biol..

[69] Zhao B (2007). Inactivation of YAP oncoprotein by the Hippo pathway is involved in cell contact inhibition and tissue growth control. Genes Dev..

[70] Aragona M (2013). A Mechanical Checkpoint Controls Multicellular Growth through YAP / TAZ Regulation by Actin-Processing Factors Mechanical Regulation of Cell Proliferation through. Cell.

[71] Sunyer, R. *et al*. Collective cell durotaxis emerges from long-range intercellular force transmission. *Science*. **353**, 1157–1161 (2016). 10.1126/science.aaf7119 27609894

[72] Nagayama K, Hamaji Y, Sato Y, Matsumoto T (2015). Mechanical trapping of the nucleus on micropillared surfaces inhibits the proliferation of vascular smooth muscle cells but not cervical cancer HeLa cells. J. Biomech..

[73] Deisenroth C, Zhang Y (2011). The ribosomal protein-mdm2-p53 pathway and energy metabolism: Bridging the gap between feast and famine. Genes and Cancer.

[74] Boulon S, Westman BJ, Hutten S, Boisvert FM, Lamond AI (2010). The Nucleolus under Stress. Mol. Cell.

[75] Bursac S (2012). Mutual protection of ribosomal proteins L5 and L11 from degradation is essential for p53 activation upon ribosomal biogenesis stress. Proc. Natl. Acad. Sci..

[76] Kapoor NR, Ahuja R, Shukla SK, Kumar V (2013). The HBx protein of hepatitis B virus confers resistance against nucleolar stress and anti-cancer drug-induced p53 expression. FEBS Lett..

[77] Jayo A (2016). Fascin Regulates Nuclear Movement and Deformation in Migrating Cells. Dev. Cell.

[78] Thomas AJ (2013). Functional mechanotransduction is required for cisplatin- induced hair cell death in the zebrafish lateral line. J. Neurosci..

[79] Zanconato F, Battilana G, Cordenonsi M, Piccolo S (2016). YAP/TAZ as therapeutic targets in cancer. Curr. Opin. Pharmacol..

[80] Chen F, Evans A, Pham J, Plosky B (2010). Cellular Stress Responses: A Balancing Act. Mol. Cell.

