# Supplementary material for: Dynamic adaptation of mesenchymal stem cell physiology upon exposure to surface micropatterns
Source: Sci Rep. 2019 Jun 24;9:9099. doi: 10.1038/s41598-019-45284-y (PMC6591423; doi:10.1038/s41598-019-45284-y)
Supplement: Supplementary file 1 — Supplementary figures [file 41598_2019_45284_MOESM1_ESM.pdf]

Supplementary figures for:

Dynamic adaptation of mesenchymal stem cell physiology upon exposure to surface micropatterns

Nick R.M. Beijer, Zarina M. Nauryzgaliyeva, Estela M. Arteaga, Laurent Pieuchot, Karine Anselme, Jeroen van de Peppel, Aliaksei S Vasilevich, Nathalie Groen, Nadia Roumans, Dennie G.A.J. Hebels, Jan de Boer

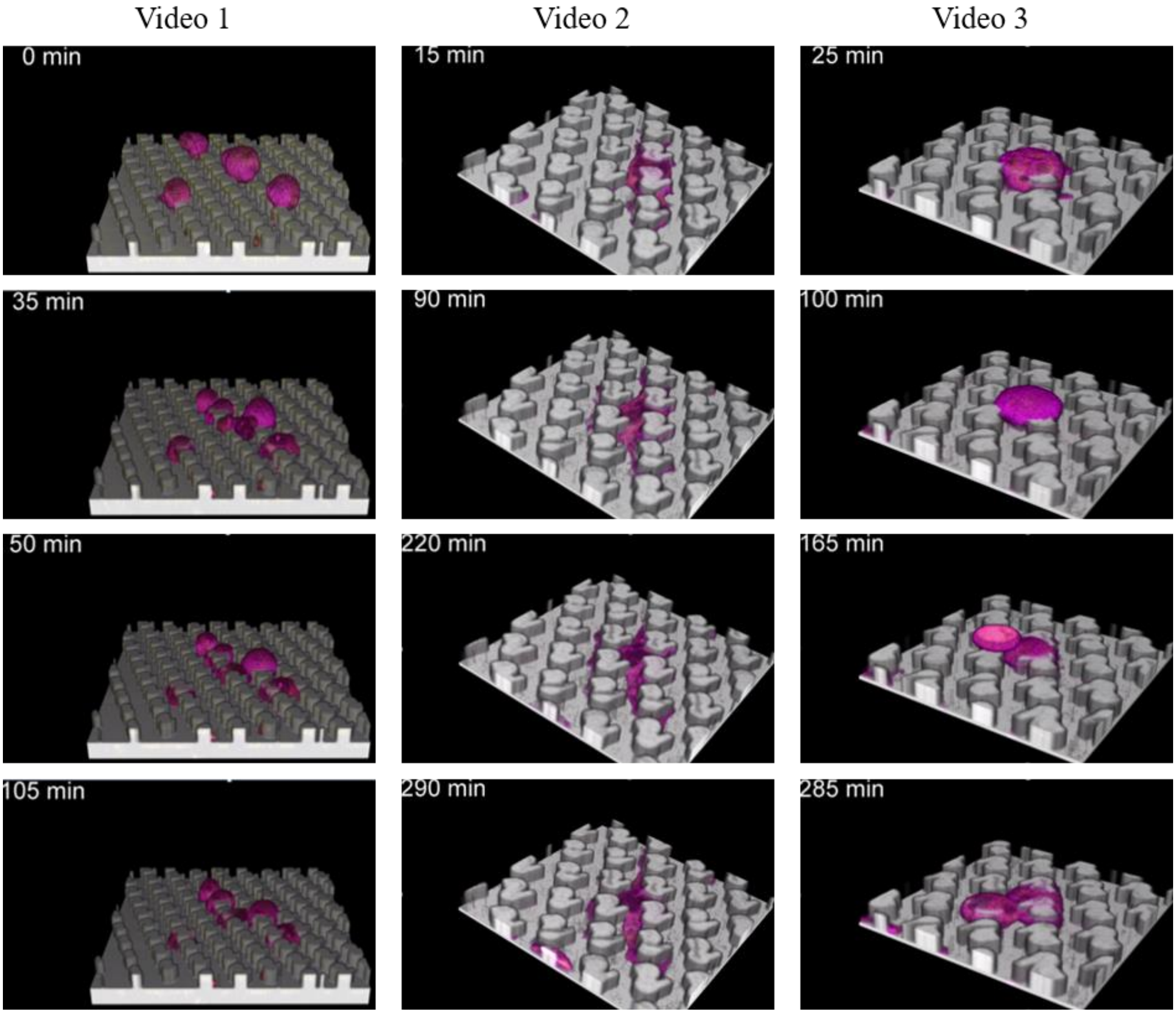

Supplementary figure 1. Cells adapting to surface topography. Selected frames of three-dimensional reconstructed confocal live cell image micrographs. Video 1 shows the attachment of fluorescently labeled U2OS cells to a topography M substrate. Video 2 shows the constant remodeling of protrusions. Video 3 shows the differences in cell-material contact during cell division. Full videos are available at www.jandeboerlab.com.

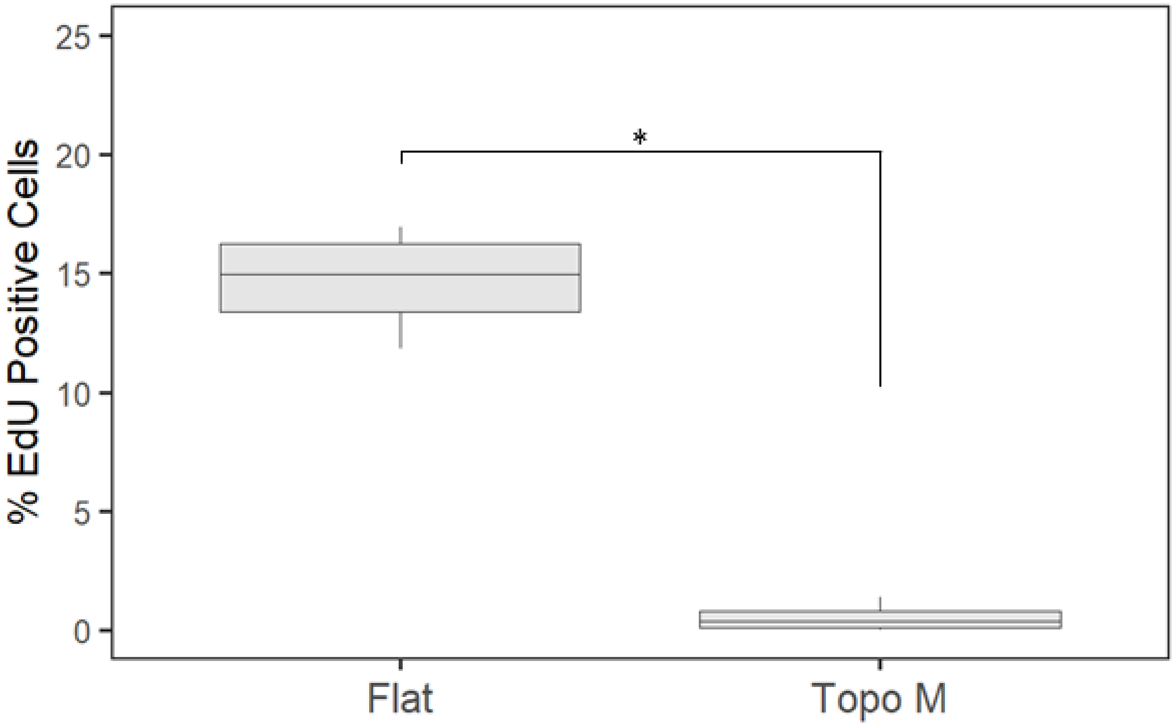

Supplementary figure 2. Validation decrease proliferation rate. A second hMSC-donor was used to validate the observation on decreased proliferation rates of hMSCs on topographically-enhanced substrates compared to flat substrates (N=4). The followed procedure was similar to the proliferation experiments from the main text using the medium size scaled topographies, however, using a female donor of 76 years old. The difference between conditions were significantly different with $p<0.05$, as indicated by the statistical significance star.
